# Adsorptive colorimetric determination of chromium(VI) ions at ultratrace levels using amine functionalized mesoporous silica

**DOI:** 10.1038/s41598-022-09689-6

**Published:** 2022-04-05

**Authors:** Rajesh Ghosh, Saranya Gopalakrishnan, T. Renganathan, S. Pushpavanam

**Affiliations:** Department of Chemical Engineering, Indian Institute of Technology Madras, Chennai, 600036 India

**Keywords:** Environmental chemistry, Sensors and biosensors

## Abstract

There is an urgent need for a rapid, affordable and sensitive analytical method for periodic monitoring of heavy metals in water bodies. Herein, we report for the first time a versatile method for ultratrace level metal detection based on colorimetric sensing. The method integrates preconcentration using a nanomaterial with a colorimetric assay performed directly on the metal-enriched nanomaterial surface. This method circumvents the need for tedious sample pre-processing steps and the complex development of colorimetric probes, thereby reducing the complexity of the analytical procedure. The efficacy of the proposed method was demonstrated for chromium(VI) ions detection in water samples. Amine functionalized mesoporous silica (AMS) obtained from a one-pot synthesis was utilized as a pre-concentration material. The structural and chemical analysis of AMS was conducted to confirm its physico-chemical properties. The pre-concentration conditions were optimized to maximise the colorimetric signal. AMS exhibited a discernible colour change from white to purple (visible to the naked eye) for trace Cr(VI) ions concentration as low as 0.5 μg L^−1^. This method shows high selectivity for Cr(VI) ions with no colorimetric signal from other metal ions. We believe our method of analysis has a high scope for de-centralized monitoring of organic/inorganic pollutants in resource-constrained settings.

## Introduction

Heavy metals are discharged into aqueous environments from various industries due to inefficient wastewater treatment facilities^1,2^. Presence of even trace concentration of these heavy metals can lead to detrimental health effects such as cancer, neurological disorder, endocrine disruption, and also trigger antimicrobial resistance (AMR)^3,4^. These problems substantiate the requirement of routine environmental monitoring of heavy metals.

Conventional methods for trace level heavy metal detection include atomic absorption spectroscopy (AAS) and inductively coupled plasma—mass spectrometry (ICP-MS). These methods are well known for their high sensitivity, selectivity, and precision. However, they require sophisticated instruments, multiple sample processing steps and trained technicians which make these methods time consuming and costly for routine environmental surveillance^5,6^.

Alternative approaches for rapid sensing of heavy metals by colorimetry^7^, fluorometry^8^ and electrochemistry^9^ have been explored. These methods offer high compatibility for integration into portable detectors, multiplex detection and high-throughput analysis^10^. Among these, colorimetric sensing is favored primarily because of its easy detection by visual colour change. Besides, it is simple, low-cost, and is rapid. It has the potential to be widely adopted for point of care applications, particularly in resource-poor settings^11^. But the major drawback of this method is its poor sensitivity to detect low concentrations (μg L^−1^) of the target analyte^12^.

This shortcoming can be overcome by incorporating preconcentration techniques. The most commonly used preconcentration technique is solid-phase extraction (SPE). In this technique, a sample is passed through a cartridge to concentrate the target analyte and eluted by passing a suitable solvent^13^. Two different approaches have been made in integrating SPE as a preconcentration technique with colorimetry. In the first approach, commercial SPE or functionalized material developed for selective enrichment, is packed in a cartridge and the enriched eluent is analysed using colorimetric indicator^14,15^. The second approach combines selective enrichment and colorimetry in a single step by impregnating an analyte specific indicator with solid-phase of the SPE cartridge for metal detection^16,17^. This method eliminates the requirement of the elution step but it requires a complex reagent impregnation process. The preconcentration process in these methods requires a very low sample flow rate to increase the retention factor making this approach time consuming. Moreover, SPE technique has limitations of adsorbent leaching, column clogging and channeling^18^.

Herein, we report a novel method for the ppb (μg L^−1^) level detection of heavy metals in aqueous samples. The proposed method termed as adsorptive colorimetry involves (a) batch adsorption for heavy metal preconcentration from a dilute solution (b) colorimetric reaction on the metal enriched adsorbent surface to obtain a color change in the visible spectrum. This method eliminates tedious pre-processing steps (conditioning, loading, washing and elution) and complex reagent impregnation and the use of sophisticated instruments. We have employed a nanomaterial for preconcentration to improve the efficacy of the proposed method. They offer high surface to volume ratio which enhances its adsorption capacity and rate^19^. In addition, they also possess higher surface atoms and unsaturated binding sites which facilitate the possibility of surface modification for selective enrichment^20^. The batch mode of operation favours effective utilization of the nanomaterial surface and enables rapid enrichment of the analyte.

To support the utility of the adsorptive colorimetric method, detection of chromium(VI) ions in μg L^−1^ level was demonstrated. Amine functionalized mesoporous silica (AMS) was prepared employing a co-condensation method for Cr(VI) ions enrichment. Morphological and chemical analysis were performed to characterize the as-synthesized AMS and to elucidate the mechanism of Cr(VI) ions adsorption. To further enhance the sensitivity of the method, adsorption parameters like contact time, pH and solution volume were optimized. Though we have focused on Cr(VI) ions detection, the scope of the proposed method is wide and it can be used for the detection of other organic/inorganic pollutants. The simplicity of the protocol permits μg L^−1^ level detection of pollutants in any general laboratory without the need of sophisticated techniques.

## Results and discussion

### Principle of adsorptive colorimetry

**Figure 1:**
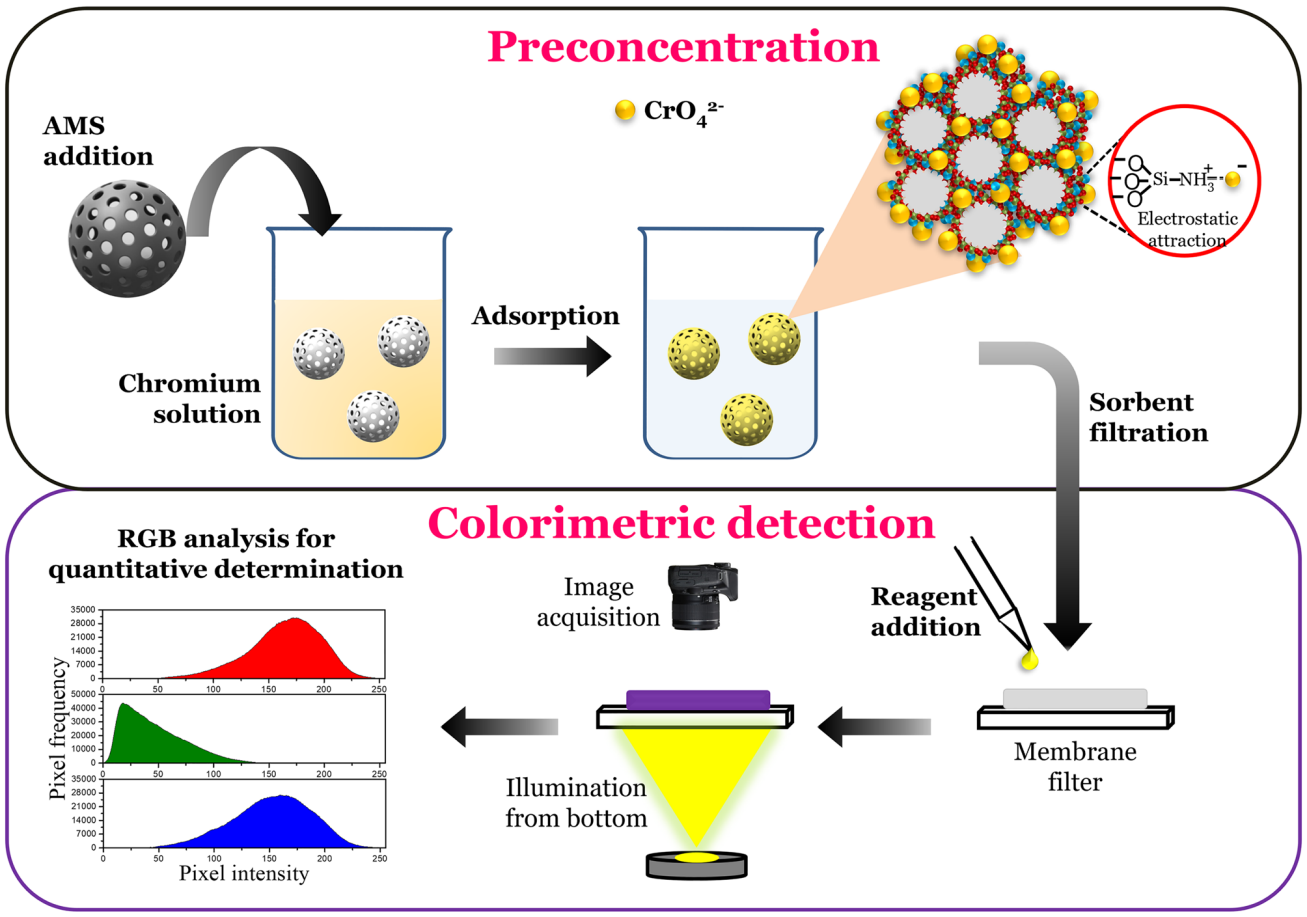
Schematic representation of the proposed method for chromium(VI) ions detection involving two steps: preconcentration of chromium(VI) ions using amine functionalized mesoporous silica followed by colorimetric detection on metal enriched AMS surface.

Figure 1 represents the working principle of adsorptive colorimetric method for chromium(VI) ions detection using amine functionalized mesoporous silica (AMS). We have carefully chosen AMS as a preconcentration nanomaterial, which has positively charged amine group for efficient enrichment of negatively charged Cr(VI) ions. The trace concentration of Cr(VI) ions in water was preconcentrated on AMS surface and the colorimetric detection using 1,5-diphenylcarbazide (DPC) reagent was performed on the metal enriched nanoparticle surface. AMS surface exhibits an intense purple coloured complex with Cr(VI) ions, which can be observed readily by naked eye. The observed colorimetric signal was recorded with portable digital detectors. RGB analysis was performed for quantitative measurement of Cr(VI) ions.

### Synthesis and characterization of amine functionalized mesoporous silica

**Figure 2:**
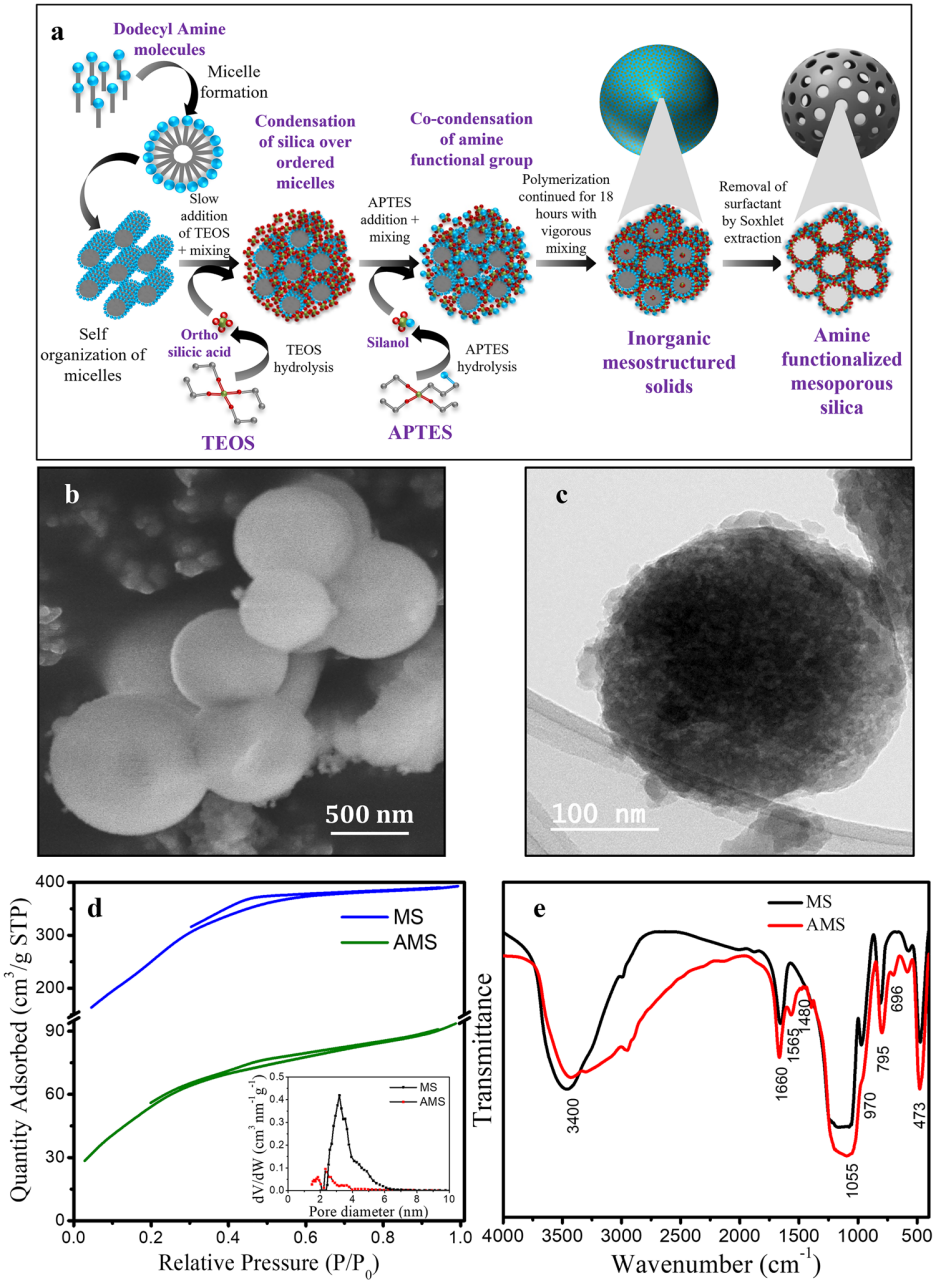
(**a**) Schematic of amine functionalized mesoporous silica synthesis using co-condensation technique (**b**) SEM images revealing spherical morphology (**c**) TEM images confirming porous nature. (**d**) Nitrogen adsorption–desorption isotherm of BET analysis for mesoporous silica without (MS) and with amine functionalization (AMS). Inset figure shows HK plot for pore size distribution (**e**) FTIR spectrum of MS and AMS.

Amine functionalized mesoporous silica (AMS) employed for the preconcentration of Cr(VI) ions was prepared using the sol–gel method^21^. Figure 2a represents the schematic of the steps involved in the synthesis of AMS. Dodecyl amine was used as a template for simultaneous polycondensation of silica and amine group. The AMS powder obtained after template removal and drying was characterized to understand its physical and chemical properties. The scanning electron microscopic (SEM) and transmission electron microscopic (TEM) images are shown in Fig. 2b,c. A spherical morphology with size ranging 200 – 800 nm was revealed from SEM and TEM analysis. The presence of porous structure could be evidenced from the TEM micrograph. The nitrogen adsorption–desorption isotherms for the particles without (MS) and with amine functionalization (AMS) are shown in Fig. 2d. The surface area of the nanoparticles was found to be 217.45 and 1047.13 m^2^ g^−1^ for the particles with and without amine functionalization respectively. Both the isotherms shown in Fig. 2d exhibits a typical Type-IV pattern which is a characteristic of mesoporous structure according to IUPAC Classification^22^. The pore size distribution of MS and AMS were analysed using Horvah-Kawazoe (HK) plot and they are shown as an inset image in Fig. 2d. The average pore diameter was found to be 2–3 nm, which falls under mesoporous structure (2–50 nm)^23^. These results support that the synthesised adsorbent is a spherical mesoporous nanoparticle.

Amine functionalization has limited the thermal stability of AMS to 100 °C as revealed from thermogravimetric analysis (Supplementary Fig. S1). Therefore, the synthesised AMS was not treated above 100 °C for any process which involved heating. No peaks were observed in the diffraction profile of AMS (Supplementary Fig. S2). This indicates that the adsorbent was disordered in nature. Typically, silica nanoparticle can be crystallized when the particle is calcined at higher temperature (> 300 °C)^24^. Calcination was not attempted in the present study since the synthesised particle has limited thermal stability.

Fourier Transform Infra-Red spectrum (FTIR) was used to identify the functional groups in MS and AMS (Fig. 2e). In both these spectra, the prominent broad absorption band at 1055–1200 cm^−1^ was assigned to asymmetric vibration of Si–O–Si group^25,26^. The peak at 473 cm^−1^ indicates the bending of O–Si–O with stretching vibrations of silanol groups^27^. The peak at 795 cm^−1^ was attributed to the bending vibrations of Si–O–Si band^25^. The peaks at 1660 and 3400 cm^−1^ were attributed to vibration of physically absorbed water molecules^28^. When compared to MS, several new peaks were found in AMS. Also, the peak near 3400 broadened which is due to N–H stretching in the range of 3200–3600 cm^−1^^29,30^. Two weak absorption bands at 696 and 1480 cm^−1^ were ascribed to bending vibrations of N–H and –NH_3_ group respectively^25,30,31^. The peak at 1565 cm^−1^ appears due to stretching and deformation of NH frequencies^30^. Moreover, the peak found at 970 cm^−1^ in MS decreased significantly in AMS which might be due to utilization of silicon hydroxyl group during amine functionalization^32^. From the FTIR spectrum, the presence of amine groups on AMS was confirmed.

**Figure 3:**
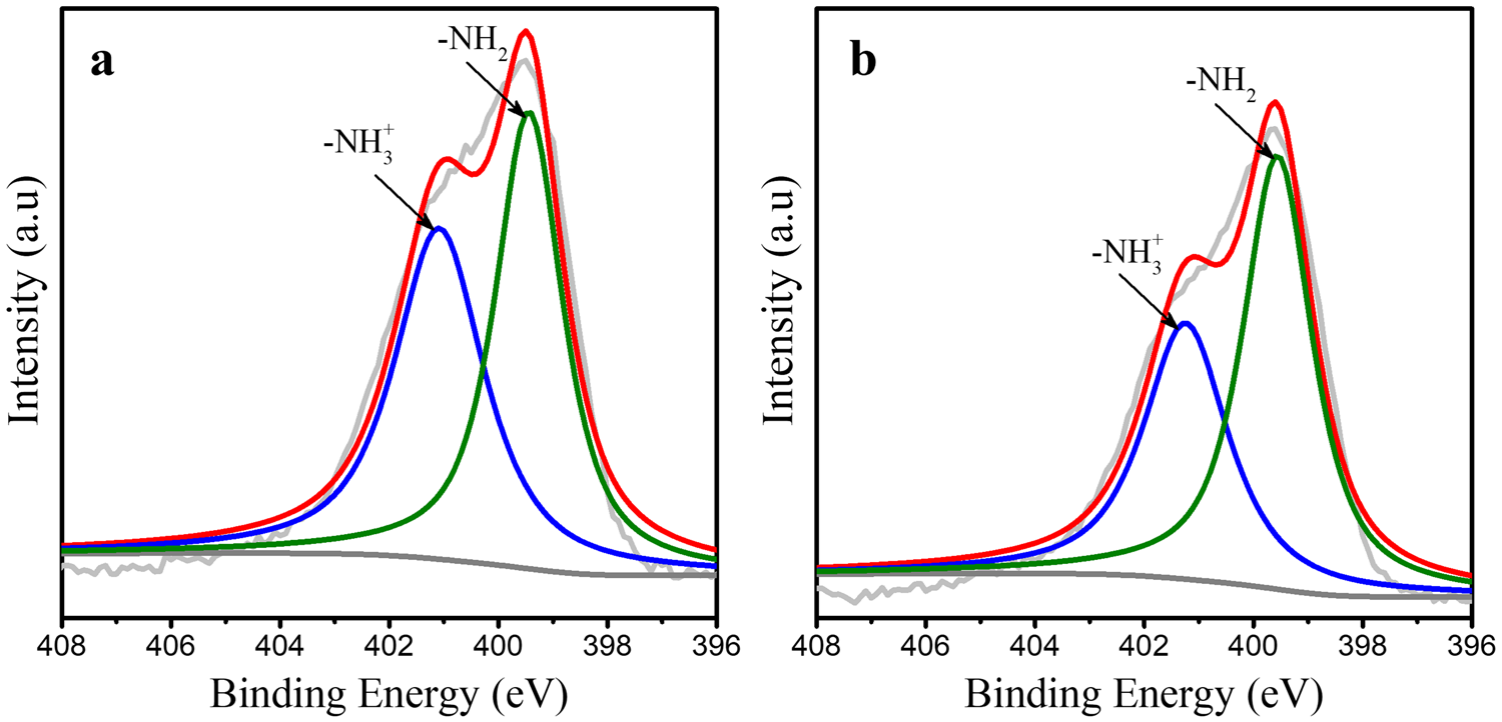
The N 1s spectra of amine functionalized mesoporous silica showing –NH_2_ and –NH_3_^+^ peaks (**a**) before adsorption (**b**) after adsorption. Decrease in –NH_3_^+^ peak was observed after adsorption.

To investigate the mechanism behind the adsorption of Cr(VI) ions on AMS, XPS analysis was performed on AMS before and after adsorption. For this, 20 mg of AMS was added to 100 ml solution of 10 µg L^−1^ Cr(VI) ions and kept in contact for 15 min. AMS was separated and analysed in XPS after drying at 50 °C. The Cr(VI) ions concentration in the filtrate solution was found to be 1.8 ± 0.8 µg L^−1^ from ICP-MS analysis. This confirmed the Cr(VI) ions adsorption on AMS surface. The core level high resolution XPS spectra were subjected to Shirley-type background subtraction to remove extrinsic loss and Gaussian–Lorentzian deconvolution was performed. Figure 3a represents the N 1s high resolution spectrum of the AMS before adsorption. The presence of primary amine group on AMS was confirmed from the FT-IR studies. The pH of the solvent (neutral) used for the synthesis and the extraction of the silica particles was lower than the acid dissociation constant of APTES (pKa = 9.73^33^). Hence, we anticipate the surface of amine functionalized silica exposes –NH_2_ and –NH_3_^+^ species. So, the N 1s spectrum was deconvoluted into two peaks at 401.1 and 399.4 eV. Peak at binding energy 401.1 eV corresponds to –NH_3_^+^ group and 399.4 eV corresponds to –NH_2_ group^31^. This confirms the presence of –NH_3_^+^ group in AMS. The N 1s spectrum of AMS after adsorption is shown in Fig. 3b. Here -NH_3_^+^ and –NH_2_ peaks were obtained at binding energy 401.5 and 399.5 eV. The binding energy shift for -NH_3_^+^ and -NH_2_ groups were found to be + 0.1 eV before and after adsorption. This insignificant change in binding energy ascertains that there is no change in electronic state. However, a substantial decrease in –NH_3_^+^ peak intensity was observed after adsorption. To understand the effect, the ratio of –NH_3_^+^/–NH_2_ was calculated from the area under the curve and found to be 96% and 71% before and after adsorption on AMS, respectively. This indicates the participation of –NH_3_^+^ group in the adsorption. Significant intensity change with insignificant binding energy shift confirms the adsorption was mainly due to electrostatic interaction between positively charged –NH_3_^+^ and anionic chromium. Moreover, zeta potential and FTIR of the AMS particles were measured before and after adsorption. It was found that the surface charge decreases from + 59.3 ± 1.8 mV before adsorption to + 51.4 ± 0.6 mV after adsorption. This establishes the electrostatic mechanism of Cr(VI) ions adsorption. Since electrostatic attraction is difficult to be detected in IR spectra^34^, no significant change in peaks of AMS was observed before and after adsorption (Supplementary Fig. S3). Also, it was found that mesoporous silica without amine functionalization (MS) showed no colour development when subjected to adsorptive colorimetric chromium(VI) ion detection. Thus, the amine functionalization plays a leading role in the Cr(VI) ions detection.

### Chromium(VI) ions detection using adsorptive colorimetry

Various preconcentration factors which influence the sensitivity of Cr(VI) ions detection such as contact time (5–60 min), solution volume (25–150 ml) and pH (2–10) of the adsorption process were investigated. All experiments were performed with 20 mg of AMS which was the minimum amount of adsorbent required to cover the entire filtration area. It was found that trans-illumination of light improved the visualization of the coloured complex when compared to epi-illumination as shown in Supplementary Fig. S4. This is because, reflectance-based measurement obtained using epi-illumination detects only the surface bound analyte Cr(VI)-DPC complex. Whereas transmittance-based measurement enables the detection of the coloured complex formed across the entire thickness of the adsorbent layer. Therefore, high sensitivity was achieved using the trans-illumination mode of image capturing.

**Figure 4:**
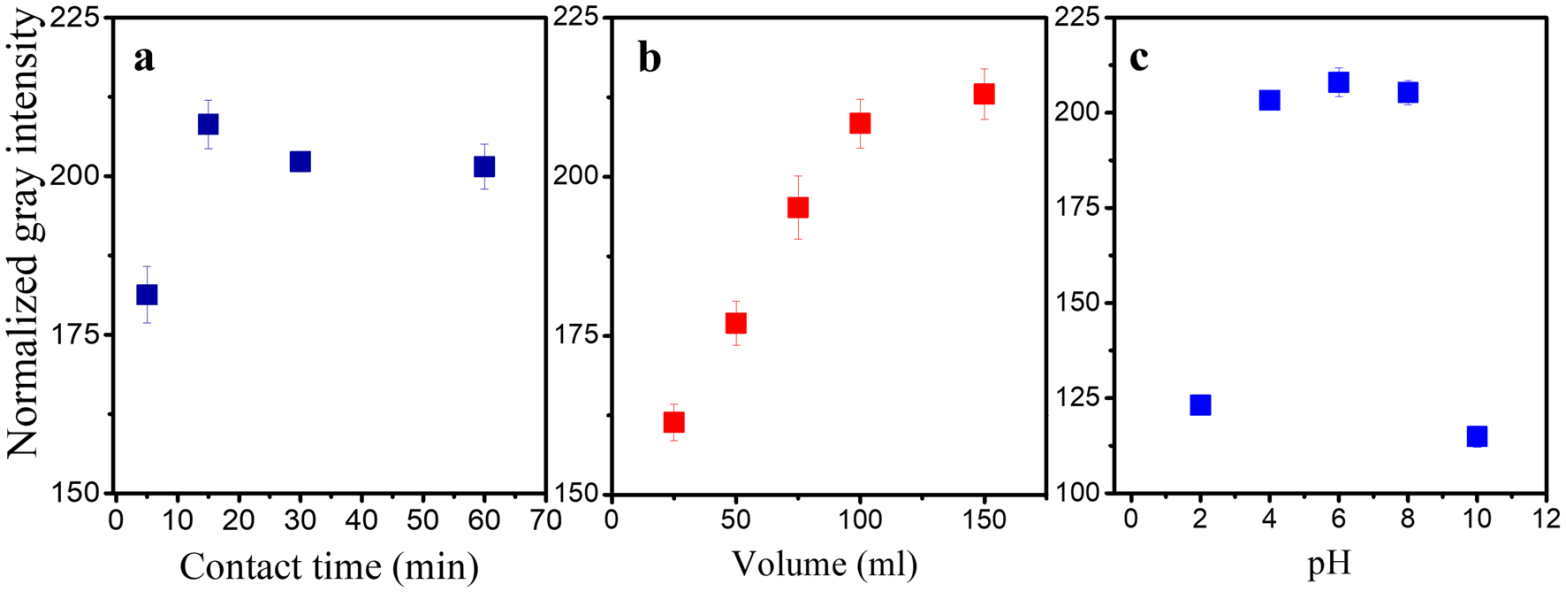
(**a**) Effect of contact time (**b**) Effect of sample volume (**c**) Effect of pH on preconcentration of chromium(VI) ions and its subsequent detection measured as normalized gray intensity.

To quantify the concentration of Cr(VI) ions, normalized gray intensity (NGI) was used as response signal and obtained from RGB analysis. The optimum preconcentration conditions were chosen based on maximum NGI obtained from adsorptive colorimetry. The effect of different contact times (5, 15, 30 and 60 min) on the adsorption and subsequent detection was studied. Figure 4a shows the normalized gray intensity after colorimetric reaction as a function of contact time. It can be observed that the Cr(VI) ions adsorption was relatively rapid. Maximum NGI was achieved in 15 min and remains almost stable till 60 min. Therefore, 15 min of contact time for adsorption was chosen for the subsequent experiments.

Effect of solution volume on the adsorption efficiency was studied and the results are shown in Fig. 4b. Chromium(VI) ions adsorption increases linearly with the solution volume till 100 mL. We hypothesize that this is due to the increase in the moles of Cr(VI) ions available for the given mass of adsorbent, with increase in the sample volume. This increases the Cr(VI) ions load on adsorbent (adsorption capacity) and aids in detection of Cr(VI) ions at very low concentration. Further increasing the solution volume to 150 mL showed only a marginal increment in NGI value compared to a solution volume of 100 mL. Hence, solution volume of 100 mL for Cr(VI) ions adsorption was selected for the calibration experiment considering the ease in handling lower sample volume.

The effect of solution pH on Cr(VI) ions detection is shown in Fig. 4c. The normalized gray intensities were found to be high for pH 4–8 and low at pH 2 and 10. Depending on the solution pH, the ionic state of the Cr(VI) ions and charge density of the adsorbent varies. At low Cr(VI) ions concentrations, typically used in this study, the Cr(VI) ions will exist as HCrO_4_^-^ and CrO_4_^2-^. Also, the amine group in the adsorbent might get protonated and exist with a higher positive charge density at neutral or acidic pH (2–8). This has more affinity to attract the Cr(VI) anions in the solution. However, since the pKa value for chromate anion is 6.4, HCrO_4_^-^ dominates at pH < 6.4, and CrO_4_^2−^ species dominates at pH > 6.4^35,36^. Therefore, at extremely acidic condition (pH 2), the monovalent HCrO_4_^−^ is present predominantly compared to its divalent CrO_4_^2−^ form. The monovalent form is hypothesized to have reduced affinity towards –NH_3_^+^ group, thereby reducing the overall Cr(VI) ions load on the adsorbent surface. On the other hand, at alkaline pH (10), adsorbent exists in deprotonated state (inactive form), which reduces the adsorption capacity of AMS. Therefore, neutral pH was chosen as the optimum pH value for further experiments.

**Figure 5:**
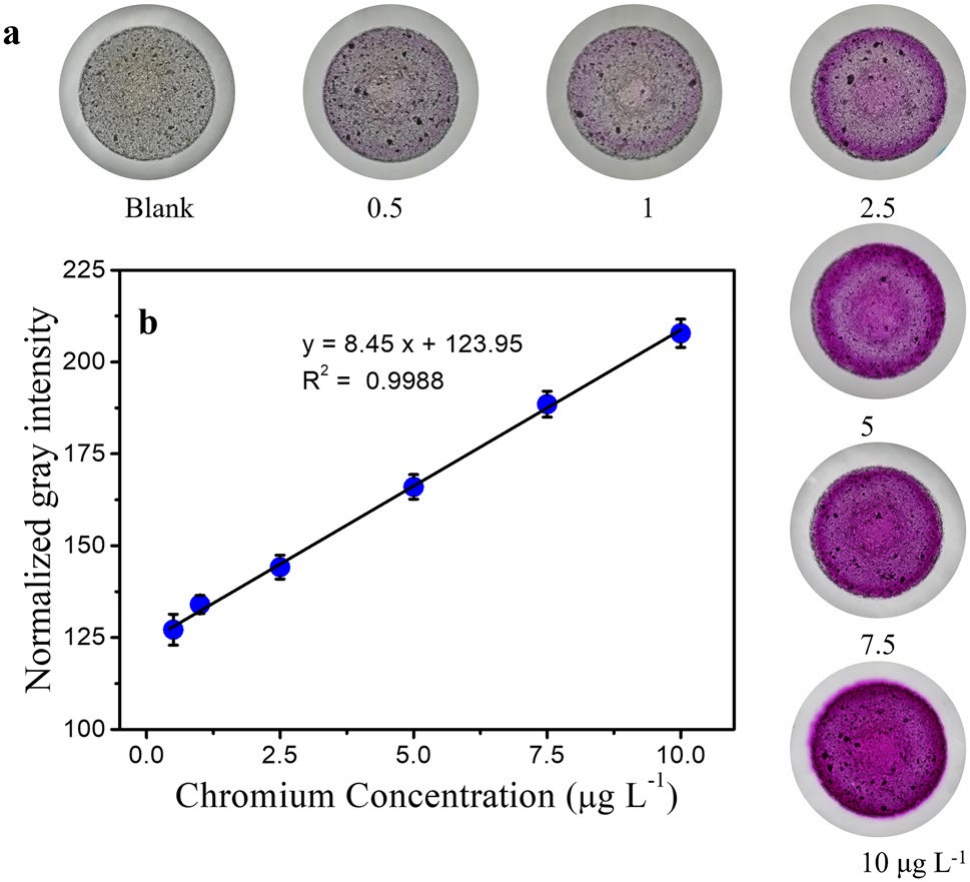
Chromium(VI) ions detection using adsorptive colorimetric method (**a**) Image of adsorbent with different concentration of Cr(VI) ions for the optimized adsorption conditions. (**b**) Linear calibration plot in the range of 0.5–10 µg L^−1^ obtained by measuring normalized gray intensity.

At the optimized adsorption conditions, adsorptive colorimetric experiments were carried out and the calibration curve for Cr(VI) ions was obtained as shown in Fig. 5. Figure 5a shows the images of adsorbent at different concentrations of Cr(VI) ions. It can be seen that the colour of adsorbent is white in the blank sample. The slight purple colour on the adsorbent was directly visible to naked eye even at 0.5 µg L^−1^ concentration. This colour intensity increases with increase in Cr(VI) ions concentration and becomes highly intense purple colour at 10 µg L^−1^ concentration. Figure 5b shows the linear calibration plot for Cr(VI) ions in the range of 0.5–10 µg L^−1^ as a function of normalized gray intensity. LOD (3.3 σ/S) and LOQ (10 σ/S) are calculated to 0.5 and 1.5 µg L^−1^ respectively where σ is the standard deviation and S is the slope of the calibration curve. The proposed method could detect 200 × and 100 × lower than permissible limit of chromium recommended by United States Environmental Protection Agency (US EPA) and Bureau of Indian Standards (BIS) for drinking water respectively^37^. Thus the proposed method of analysis opens up the scope for periodic environmental monitoring of Cr(VI) ions even at very low concentrations.

**Figure 6:**
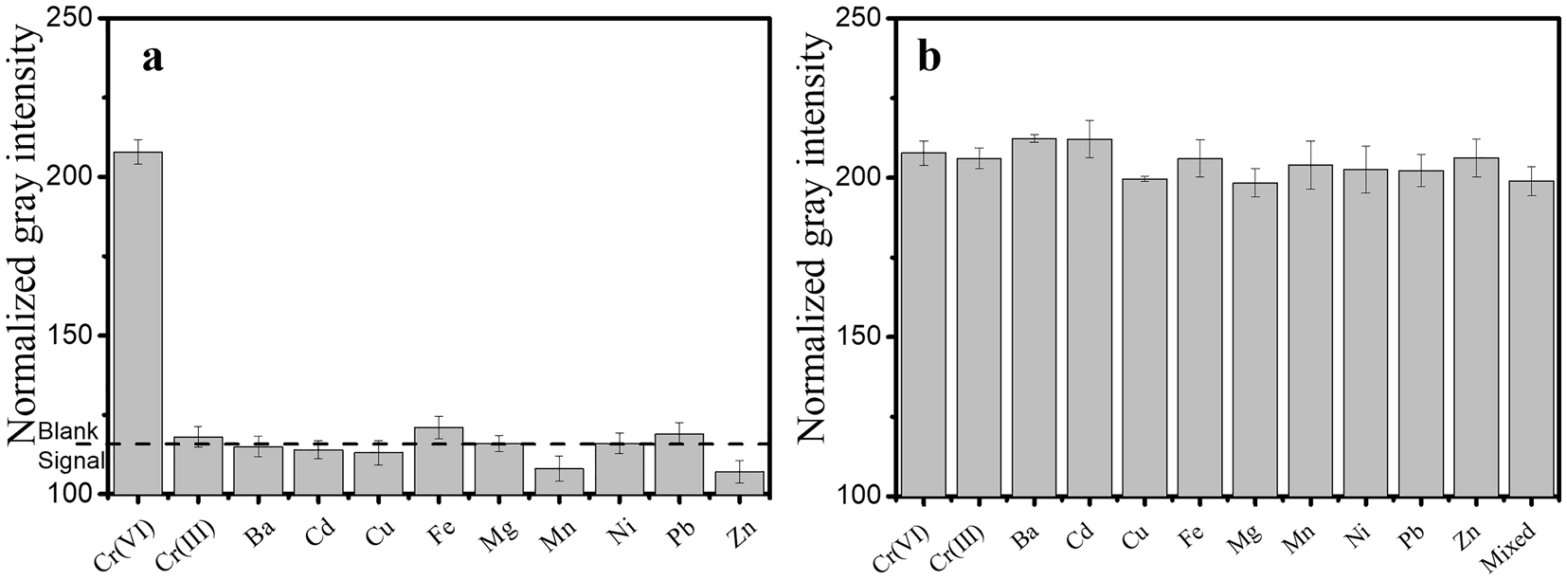
(**a**) Normalized gray intensities of chromium(VI) ions (10 µg L^−1^) and different metal ions (100 µg L^−1^) at the optimized adsorptive colorimetric reaction conditions. Blank signal represents background normalized gray intensity. (**b**) Normalized gray intensities of mixtures of chromium(VI) ions (10 µg L^−1^) with different metal ions (each at 100 µg L^−1^). Mixed indicates 10 µg L^−1^ each of all metal ions.

Interference of different metal ions (Cr(III), Ba, Cd, Cu, Fe, Mg, Mn, Mg, Ni, Pb and Zn) was studied to test the selectivity of the adsorptive colorimetric method. Solutions containing each of the different metal ions were prepared at 100 µg L^−1^ concentration and subjected to adsorptive colorimetry without and with Cr(VI) ions at 10 µg L^−1^. Figure 6a,b show the normalized gray intensity for these different metal ion solutions without and with Cr(VI) ions respectively. It can be seen that from Fig. 6a that all the normalized gray intensities were close to that of the blank signal. The reaction of diphenyl carbazide is reported to be nearly specific to Cr(VI) ions. Iron may produce a yellow colour at high concentration (1 mg L^−1^)^38^. However, in the present study iron interference was not observed. This may be attributed to the incorporation of adsorption for preconcentration thereby increasing the selectivity of the adsorptive colorimetric technique. More importantly, it can be observed that the proposed method selectively detects highly toxic Cr(VI) ions at ultra-trace concentrations in the presence of relatively non-toxic Cr(III) ions. Figure 6b shows that the colorimetric signal from Cr(VI) in the presence of interfering ions was almost equivalent to that of Cr (VI) ions alone. Also, it was found that co-existence of all metal ions (each at 10 µg L^−1^) did not interfere in Cr(VI) ions determination. These findings confirm the selectivity of the proposed method for Cr(VI) ions detection.

Consumption of chromium (VI) through drinking water was reported to be clear carcinogenic in animal studies^39^. Hence, adsorptive colorimetric method was applied to drinking water samples. Table 1 shows the comparison of Cr(VI) ions measurement in the spiked drinking water samples by standard ICP-MS and adsorptive colorimetric method. Relative recoveries (RR) of Cr(VI) ions were found to be ≥ 90% which indicates the high accuracy of the proposed method. Also, the relative standard deviation of the proposed method was found to be < 6% which reveals the high precision of the adsorptive colorimetric method. Therefore, the proposed adsorptive colorimetric method could detect Cr(VI) ions up to parts per billion (ppb) levels in water sample successfully without the need of sophisticated analytical tool.

**Table 1:** Analysis of water samples spiked with Cr(VI) ions using adsorptive colorimetry and ICP-MS method.

Sample	Spiked (µg L^−1^)	Chromium(VI) ions concentration (µg L^−1^)				
		Current method	RSD (%)	ICP-MS	RSD (%)	RR (%)
I	5	5.33	5.95	5.94	2.45	89.6
II	10	10.05	5.26	10.29	1.17	97.6

### Comparison with commercial mesoporous silica

The amine functionalized mesoporous silica synthesized in the present study using co-condensation method could achieve trace level detection of Cr(VI) ions. To compare the efficacy of AMS, commercially available mesoporous silica (SBA-15) was amine functionalized by post grafting method^40^. The functionalization protocol is given in Supplementary section S-1. Adsorptive colorimetric ability of amine functionalized SBA-15 (A-SBA) was tested. A slight purple colour was observed in A-SBA with 10 µg L^−1^ Cr(VI) ions concentration. Estimation of normalized gray intensity revealed < 10% signal for A-SBA compared to that of AMS. To investigate the reason behind this decreased sensitivity, XPS analysis was performed for A-SBA. N 1s spectrum of A-SBA is shown in Supplementary Fig. S5. This reveals a negative 0.8 eV shift in binding energy for –NH_3_^+^ peak (400.3 eV) when compared to AMS (401.1 eV). Decrease in binding energy could be due to decrease in positive charge or increase in electron density around the atom^41^. Thus, A-SBA has relatively low positive charge on the surface and this resulted in reduced signal from adsorptive colorimetric assay. Also, the ratio of –NH_3_^+^/–NH_2_ for A-SBA (70%) was 26% lower than that of AMS (96%). Moreover, amine functionalized mesoporous silica synthesized using co-condensation method was reported to have homogenous amine functionalization on both internal and external surface of bulk silica particle^42^. Hence synthesized AMS showed superior adsorptive colorimetric signal when compared to amine grafted SBA-15.

### Adsorptive colorimetry compared with existing methods

Table 2 shows the comparison of the proposed method with the other existing colorimetric methods available for the detection of Cr(VI) ions. The existing colorimetric methods can detect Cr(VI) ions in the range of ppm with the use of spectrophotometric equipment. Compared with the existing paper-based microfluidics devices/test strips, the proposed method showed high sensitivity with LOD of 0.0005 mg L^−1^, which is very much lower than the existing methods. The proposed method has potential to be used in point of care application with hand-held devices for adsorbent removal and portable camera for image acquisition. It overcomes the need for sophisticated instruments and complex pre-processing procedure with the conventional analysis techniques. A simple adsorbent based preconcentration without the need for elution step and direct colorimetric assay on the adsorbent surface forming chromophore, enables rapid and sensitive determination of the analyte. However, the technique is limited to non-turbid and uncoloured samples. Also the proposed method requires white based adsorbent to perform colorimetric assay.

**Table 2:** Comparison of existing methods with adsorptive colorimetric method for the detection of Cr(VI) ions.

Method	Detection	Reagent	Range (mg L^−1^)	LOD (mg L^−1^)	References
Spectrophotometer	Colorimeter	DPC	0.2–1	–	^43^
Paper based microfluidics	Colorimeter	DPC	40–400	30	^44^
Paper based microfluidics	Colorimeter	Gold nanoparticle-silanization-titanium dioxide	0.026–2.6	0.014	^45^
Rotational Paper based microfluidics	Colorimeter	DPC	0.5–10	0.18	^46^
Paper test strip	Photoluminescence	Glutathione capped- gold nanoclusters	0.03–0.83	0.03	^47^
Adsorptive colorimetric method	Colorimeter	DPC	0.0005–0.010	0.0005	Present study

## Conclusions

We propose a simple, rapid and affordable method to quantify contaminants in ppb levels with integration of batch adsorption and colorimetric detection. Adsorptive colorimetric determination of Cr(VI) ions was illustrated using amine functionalized mesoporous silica (AMS) as adsorbent. AMS was synthesised by one-pot method and the characterization reveals that synthesised silica particle was spherical, mesoporous in nature and amine functionalized. Chromium(VI) ions adsorption mechanism of AMS was elucidated with XPS analysis. The ability of AMS to achieve Cr(VI) ions detection with integration of 1,5-Diphenyl carbazide colorimetric assay was demonstrated to be highly selective and sensitive (0.5–10 µg L^−1^). The method showed high accuracy and precision for the spiked drinking water samples in ppb levels. There is a striking positive signal for Cr(VI) ions presence by synthesized AMS compared to amine functionalized SBA-15 (synthesized by post-grafting method) under similar conditions.

Batch-adsorption based pre-processing provides a simple platform for preconcentration of analyte and integration with colorimetric detection on the adsorbent surface facilitates the detection with lower complexity and enhanced sensitivity. Adsorption, filtration and image acquisition process can be performed using hand-held devices and portable camera. The present method does not need any sophisticated instrument or tedious process and hence can potentially be used for point of care application especially in resource-constrained areas. The distinct features of this proposed method are simplicity, low cost, field deployable without compromising sensitivity.

## Materials and methods

### Chemicals

Dodecyl amine (98%), tetraethyl orthosilane (TEOS, 98%) and 3-aminopropyl triethoxysilane (APTES, 99%) were purchased from Sigma Aldrich. AR grade 1,5- Diphenylcarbazide (DPC 98%) was obtained from Loba Chemie. Sulphuric acid (98%) and ethanol (99%) were obtained from Rankem. Potassium dichromate (99%), 0.1 N Hydrochloric acid, 0.1 N sodium hydroxide were purchased from Merck. Silica, Mesoporous SBA-15 (< 150 µm particle size and 450–550 m^2^ g^−1^ surface area, Catalogue number: 806854) was obtained from Sigma Aldrich. Lead nitrate, Copper sulphate pentahydrate, Ammonium ferrous sulphate hexahydrate, Nickel sulphate pentahydrate, Manganese sulphate monohydrate, Chromium(III) nitrate nonahydrate, Magnesium sulphate heptahydrate, Cadmium nitrate heptahydrate, Zinc nitrate hexahydrate, Barium chloride dihydrate were purchased from Merck. All experiments were conducted using ultrapure water obtained from a MilliQ system.

### Synthesis of amine functionalized mesoporous silica

Amine functionalized mesoporous silica (AMS) was synthesized by the co-condensation method^21^. Briefly, 0.25 mol of dodecyl amine was dissolved in a mixture of 10 mol and 50 mol of ethanol and deionised water, respectively. To this mixture, 1 mol of TEOS was added dropwise under constant stirring. After 30 min of addition, 0.25 mol of APTES was added and the mixture was stirred for 18 h at room temperature. The suspended particles were separated from the mixture using a 47 mm diameter vacuum filtration setup (0.45 µm pore size nylon filter paper) and washed thoroughly with deionised water. The residue was dried at room temperature for 24 h. For the removal of APTES and dodecyl amine, continuous extraction using a soxhlet apparatus was carried out with ethanol for 72 h. Silica particles were then dried at 50 °C for three hours. The particles were then grounded and vacuum dried at 100 °C for 8 h. Mesoporous silica without amine functionalization (MS) was synthesised under same conditions without the addition of APTES.

### Characterization of AMS

Synthesized AMS were characterized using scanning electron microscope (SEM) and transmission electron microscope (TEM) for morphological analysis. SEM (HR-SEM, Hitachi S4800, accelerating voltage of 5 kV) was used to determine particle morphology. TEM was done using Tecnai T12 TEM at an accelerating voltage of 200 kV to determine particle size and morphology. Brunauer–Emmett–Teller (BET) analysis (Micromeritics ASAP 2020 Porosimeter) was performed to obtain its surface area and characterize its porosity. For this, the material was degassed at 100 °C overnight before obtaining the nitrogen adsorption–desorption isotherms. Thermogravimetric analysis was used to determine thermal stability of the adsorbent. The sample was heated from room temperature to 900 °C at a rate of 10 °C min^−1^ under a nitrogen atmosphere and the analysis was performed using Q500 Hi-Res TGA instrument. Powder X-ray Diffraction (XRD) was used for phase identification and was carried out using D8 Advance (Bruker) with 2θ scanned in the range of 0° to 10°. Fourier transform infrared (FTIR spectrum) was obtained by Bruker IFS66v FT-IR with a frequency range 4000 cm^−1^ to 400 cm^−1^ and resolution of 1 cm^−1^ to identify the functional groups present in MS and AMS. X-ray photoelectron spectroscopy (XPS) was performed on a Thermo Fisher Scientific ESCALAB Xi + instrument with Al K-alpha micro-focused monochromatic X-ray source to analyse the elemental composition and chemical nature of the material. The binding energy was standardized to C 1s adventitious carbon at 284.8 eV. Zeta Potential measurements were conducted using Horiba-SZ-100 instrument. For this, AMS particles were uniformly dispersed in ultrapure water (0.01% w/v) using an ultrasonic bath. The surface charge of the particle was measured at pH 7.

### Adsorptive colorimetric method

Adsorption experiments using amine functionalized mesoporous silica were conducted under batch conditions. For this a solution of predefined Cr(VI) ions concentration was contacted with the adsorbent for a defined contact time in a rotary shaker (250 rpm, Remi RS18Plus). The adsorbent was then separated using a vacuum filtration setup (Borosil, 25 mm Glass filtration setup) with membrane filter (0.45 µm nylon 25 mm filter paper, Pall Corporation). The effective filtration diameter of the filtration setup was 14 mm. The adsorbent along with the filter paper was dried at 50 °C for 5 min in a hot air oven.

Colorimetric detection of Cr(VI) ions was performed by addition of 1,5-Diphenyl carbazide under acidic conditions directly on the surface of the adsorbent. The reagent was prepared by mixing equal volumes of DPC (5 mg in 50% ethanol) and 2 N sulphuric acid. DPC reacts with Cr(VI) ions under acidic conditions and forms diphenyl carbazone (DPCA), a purple-coloured complex as described by the reaction given in Eq. (1).

1 $${\text{2CrO}}_{4}^{2 - } + \begin{array}{ll} {{\text{3C}}_{{{13}}} {\text{H}}_{{{14}}} {\text{N}}_{{4}} {\text{O}} + {\text{8H}}^{ + } } \\ {{\text{Diphenylcarbazide}}(DPC)\,{\text{colorless}}} \\ \end{array} \longrightarrow \begin{array}{ll} {\left[ {{\text{Cr}}^{{ + {3}}} \left( {{\text{C}}_{{{13}}} {\text{H}}_{{{12}}} {\text{N}}_{{4}} {\text{O}}} \right)_{{2}} } \right]^{ + } } \\ {{\text{Cr}}\left( {{\text{III}}} \right) - {\text{Diphenylcarbazone}}\;{\text{complex}}\;\left( {{\text{DPCA}}} \right)\;{\text{Purple}}} \\ \end{array}$$

The formation of purple complex occurred instantaneously, and all the images were acquired after five minutes to allow for complete colour development^38^. Images were captured using a DSLR camera (Panasonic DMC-GH4) in trans-illumination mode by passing white LED light (15 W, Milo series by Litaski) from the bottom of the filter paper and processed using ImageJ software to obtain RGB (Red, Green and blue intensity) values. The normalized gray intensity was calculated using Eq. (2)^48^.

2 $$Normalize\, gray\,intensity=255-(0.2126 R+0.7152 G+0.0722B)$$

Effects of contact time, solution volume and pH on adsorption were studied and the normalized gray intensities obtained were used to optimize these adsorption parameters. An initial Cr(VI) ions concentration of 10 μg L^−1^ was used for these optimization experiments. Adsorption experiments were conducted at different contact time of 5, 15, 30 and 60 min to determine the optimum time required to achieve maximum normalized gray intensity signal. 20 mg of AMS added to each Cr(VI) ions solution of volume of 25, 50, 75, 100 and 150 ml for a contact time of 15 min was used to investigate the effect of sample volume. Effect of pH (2, 4, 6, 8 and 10) on Cr(VI) ions adsorption was studied with 100 ml sample volume. The solution pH was adjusted using 0.1 N HCl/0.1 N NaOH measured using Oakton pH 700 benchtop pH meter. The optimized conditions were used to get calibration model. All experiments were done in triplicates and the mean normalized gray intensity is reported in the results section. Interference studies were carried out with different metal ion solutions (Cr(III), Ba, Cd, Cu, Fe, Mg, Mn, Mg, Ni, Pb and Zn). Solutions of different metal ions were taken at 100 µg L^−1^ concentration. The proposed method was applied to the solution of these individual metal ions without and with Cr(VI) ions. Experiment with all metal ions each at 10 µg L^−1^ along with Cr(VI) ions at 10 µg L^−1^ was also conducted. Experiment without any metal ions (de-ionized water) was used to obtain background NGI.

The proposed method was used for the analysis of Cr(VI) ions concentration in simulated drinking water sample. For this, 500 ml of drinking water samples were spiked with two different concentrations of Cr(VI) ions (5 and 10 µg L^−1^). The samples were analysed by proposed technique, ICP-MS and the relative recovery (%) with respect to standard method was calculated as given in Eq. (3). Experiments were triplicated and average relative recovery along with relative standard deviation is reported.

3 $$Relative\,recovery \left(\%\right)= \frac{{C}_{adsorptive\,colorimetry}}{{C}_{ICP-MS}} \times 100$$

## Supplementary Information

Supplementary Information.
