# Supplementary material for: Adsorptive colorimetric determination of chromium(VI) ions at ultratrace levels using amine functionalized mesoporous silica
Source: Sci Rep. 2022 Apr 5;12:5673. doi: 10.1038/s41598-022-09689-6 (PMC8983689; doi:10.1038/s41598-022-09689-6)
Supplement: Supplementary file 1 — Supplementary Information. [file 41598_2022_9689_MOESM1_ESM.pdf]

ADSORPTIVE COLORIMETRIC DETERMINATION OF CHROMIUM(VI) IONS AT ULTRATRACE LEVELS USING AMINE FUNCTIONALIZED MESOPOROUS SILICA

Rajesh Ghosh [1], Saranya Gopalakrishnan[1], T. Renganathan[1], S. Pushpavanam[1*]

[1]Department of Chemical Engineering, Indian Institute of Technology Madras, Chennai, 600036, India

*Correspondence and request for materials should be addressed to S.P. (E-mail: spush@iitm.ac.in)

Supplementary Information

Thermal stability analysis:

Thermal stability of the synthesized silica was studied by thermogravimetric analysis (TGA). The thermogram for the amine functionalized mesoporous silica is shown in Figure S1. It has been reported that the amine group decomposes in the temperature range 200-700 ^{o}C and amine functionalization of mesoporous silica showed a 20% weight loss[1]. The AMS synthesized in this work also showed similar weight loss of 21% till 700 ^{o}C.

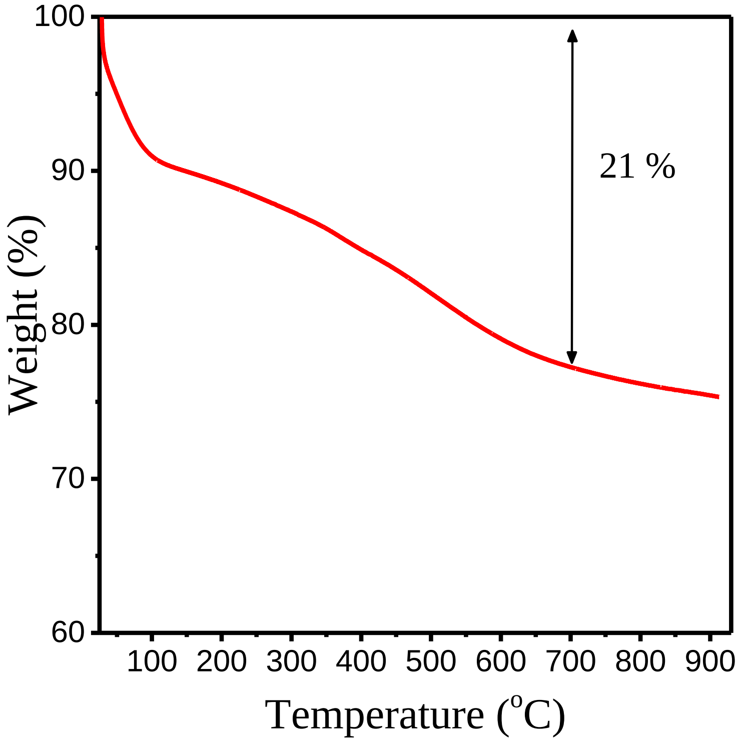

Figure S1: Thermogram of amine functionalized mesoporous silica (AMS).

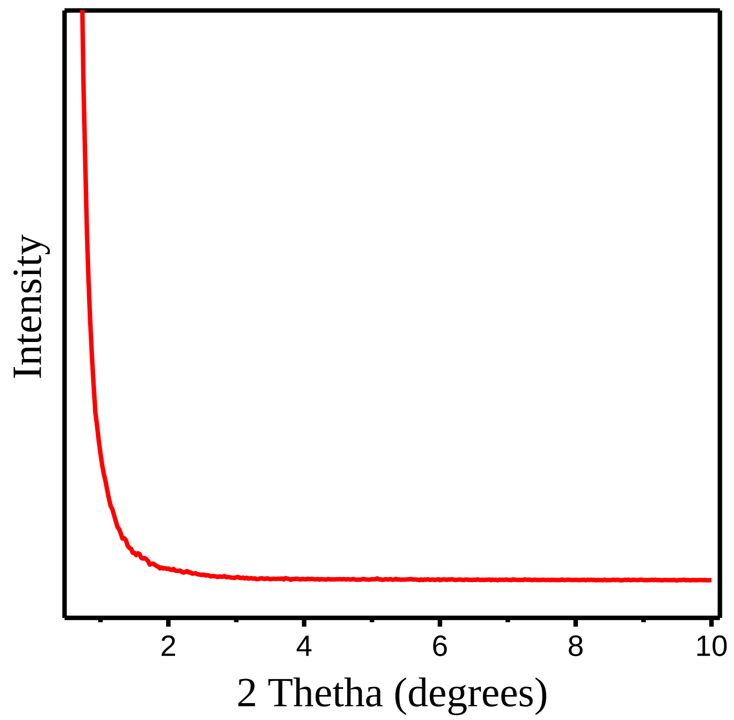

Figure S2: Powder X-ray diffraction (XRD) of amine functionalized mesoporous silica (AMS). No peak indicates the amorphous nature of AMS.

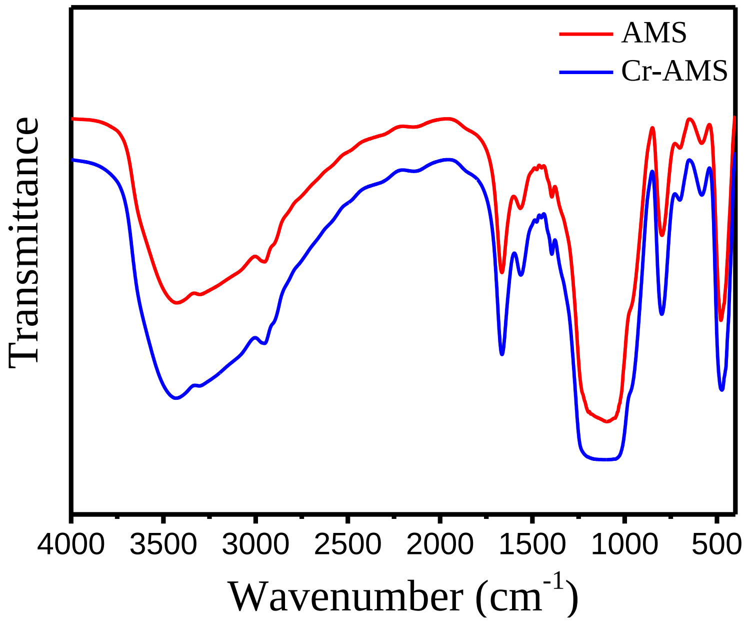

Figure S3: FTIR spectra of amine functionalized mesoporous silica before (AMS) and after Cr(VI) ions (Cr-AMS) adsorption

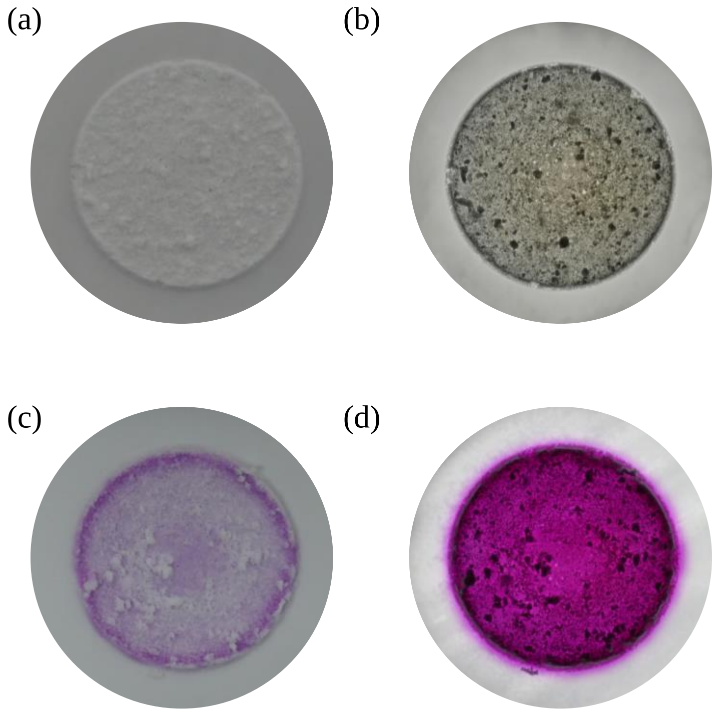

Figure S4: Effect of trans-illumination and epi-illumination on detection of Cr(VI) ions (a) epi-illumination for blank (b) trans-illumination for blank (c) epi-illumination for 10 μg L^{-1} Cr(VI) ions sample (d) trans-illumination for 10 μg L^{-1} Cr(VI) ions sample

S1: Amine functionalization of SBA-15 Functionalization of SBA-15 by APTES (3-aminopropyl triethoxysilane) was carried out as reported in previous method with slight modification[2]. The concentration of APTMS (0.25M) which is equivalent to concentration used in AMS synthesis was chosen. 0.25 M of APTES dissolved in 50 ml of ethanol was refluxed at 70 ^{0}C for 2 hours. 0.5 g of SBA-15 were loaded into APTES solution and refluxed for 6 hours. The silica particle was washed with ethanol thrice and dried at 50 ^{0}C overnight. The particle was grounded and vacuum dried at 100 ^{0}C for 8 hours to obtain amine functionalized SBA-15 (A-SBA).

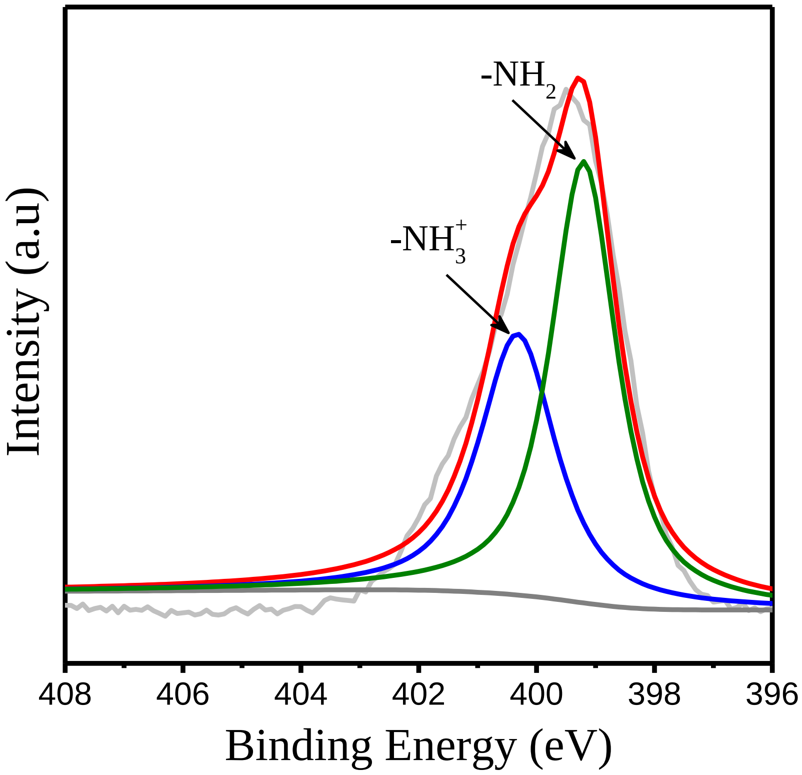

Figure S5: N 1s spectrum of amine functionalized SBA-15 by post-grafting method

References:

1. Ahmadi, E., Dehghannejad, N., Hashemikia, S., Ghasemnejad, M. & Tabebordbar, H. Synthesis and surface modification of mesoporous silica nanoparticles and its application as carriers for sustained drug delivery. *Drug Deliv.* **21**, 164–172 (2014).

2. Albayati, T. M., Salih, I. K. & Alazzawi, H. F. Synthesis and characterization of a modified surface of SBA-15 mesoporous silica for a chloramphenicol drug delivery system. *Heliyon* **5**, e02539 (2019).
